# Resveratrol Attenuates Aflatoxin B_1_-Induced ROS Formation and Increase of m^6^A RNA Methylation

**DOI:** 10.3390/ani10040677

**Published:** 2020-04-13

**Authors:** Jiamin Wu, Zhending Gan, Ruhao Zhuo, Lili Zhang, Tian Wang, Xiang Zhong

**Affiliations:** College of Animal Science and Technology, Nanjing Agricultural University, No. 6, Tongwei Road, Xuanwu District, Nanjing 210095, China; 2018105066@njau.edu.cn (J.W.); 2017105067@njau.edu.cn (Z.G.); 2019105076@njau.edu.cn (R.Z.); zhanglili@njau.edu.cn (L.Z.); twang18@163.com (T.W.)

**Keywords:** resveratrol, aflatoxin B_1_, reactive oxygen species, m^6^A RNA methylation

## Abstract

**Simple Summary:**

Aflatoxin B_1_ (AFB_1_) is highly hepatotoxic in both animals and humans. Resveratrol, a naturally-occurring polyphenolic compound, has antioxidative, anti-inflammatory, antiapoptotic, and immunomodulatory functions and plays a critical role in preventing liver damage. However, whether N^6^-methyladenosine (m^6^A) mRNA methylation, which plays critical roles in regulating gene expression for fundamental cellular processes, is associated with the protective effects of resveratrol in attenuating aflatoxin B_1_ induced toxicity is unclear. Here, we found that AFB_1_-induced reactive oxygen species (ROS) accumulation changed m^6^A modification, and the role of resveratrol in alleviating the effect on hepatic disorder induced by aflatoxin B_1_ may be due to the removal of ROS, followed by the decreased abundance of m^6^A modification, and ultimately exerting its protective role in the liver. Together, this work provides key insights into the potential avenues for the treatment of AFB_1_-induced hepatotoxicity and other relevant liver diseases.

**Abstract:**

Aflatoxin B_1_ (AFB_1_) is one of the most dangerous mycotoxins in both humans and animals. Regulation of resveratrol is essential for the inhibition of AFB_1_-induced oxidative stress and liver injury. Whether N^6^-methyladenosine (m^6^A) mRNA methylation participates in the crosstalk between resveratrol and AFB_1_ is unclear. The objective of this study was to investigate the effects of AFB_1_ and resveratrol in m^6^A RNA methylation and their crosstalk in the regulation of hepatic function in mice. Thirty-two C57BL/6J male mice were randomly assigned to a CON (basal diet), RES (basal diet + 500 mg/kg resveratrol), AFB_1_ (basal diet + 600 μg/kg aflatoxin B_1_), and ARE (basal diet + 500 mg/kg resveratrol and 600 μg/kg aflatoxin B_1_) group for 4 weeks of feeding (*n* = 8/group). Briefly, redox status, apoptosis, and m^6^A modification in the liver were assessed. Compared to the CON group, the AFB_1_ group showed increased activities of serum aspartate aminotransferase (AST) and alanine aminotransferase (ALT), prevalent vacuolization and cell edema, abnormal redox status, imbalance apoptosis, and especially, the higher expression of cleaved-caspase-3 protein. On the contrary, resveratrol ameliorated adverse hepatic function, via increasing hepatic antioxidative capacity and inhibiting the expression of cleaved-caspase-3 protein. Importantly, we noted that reactive oxygen species (ROS) content could be responsible for the alterations of m^6^A modification. Compared to the CON group, the AFB_1_ group elevated the ROS accumulation, which led to the augment in m^6^A modification, whereas dietary resveratrol supplementation decreased ROS, followed by the reduction of m^6^A levels. In conclusion, our findings indicated that resveratrol decreased AFB_1_-induced ROS accumulation, consequently contributing to the alterations of m^6^A modification, and eventually impacting on the hepatic function.

## 1. Introduction

Aflatoxins (AF) are naturally produced by *Aspergillus flavus* and *Aspergillus parasiticus*, contaminating a wide range of agricultural products, including corn, peanuts, and soybeans, due to poor storage conditions or natural disasters, and thereby harming animal and human health [1]. Among about twenty aflatoxins found, AFB_1_ is listed as a first-grade carcinogen because of its violent toxicity, intensive harm, and widespread distribution [2]. AFB_1_ is a potent hepatotoxic and hepatocarcinogenic mycotoxin. AFB_1_ is metabolized by the cytochrome P-450 enzyme system to produce AFB_1_-exo-8,9-epoxide (AFBO) in the liver, which covalently binds biomacromolecules such as DNA, RNA, and proteins in hepatocytes [3,4,5], inducing oxidative stress and hepatocyte apoptosis [6], eventually leading to liver damage and even cancer. Therefore, the hepatic toxicity of AFB_1_ to humans, poultry, and livestock and the resulting degradation of quality in animal husbandry products has drawn great attention.

**Figure 1:**
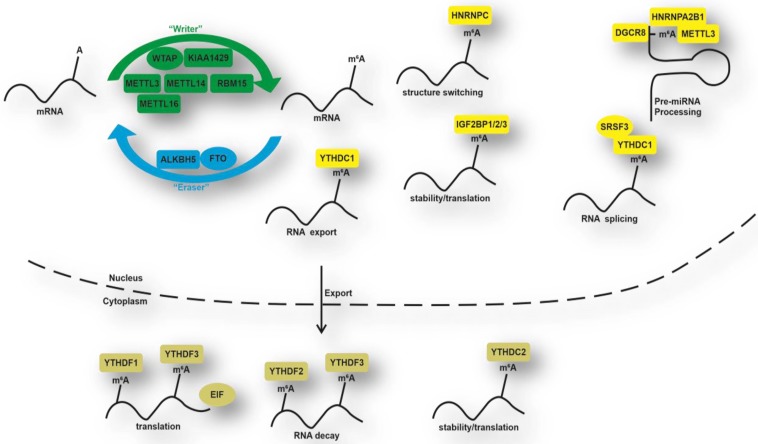
Summary of m^6^A modification machinery. N^6^-methyladenosine (M^6^A) is catalyzed by methylases, which serve as ‘writers’ containing methyltransferase-like 3 (METTL3), methyltransferase-like 14 (METTL14), wilms’ tumor 1-associating protein (WTAP), and a series of additional subunits. Fat mass and obesity-associated protein (FTO) and alkB homolog 5 (ALKBH5) serve as ‘erasers’ and exert a demethylation function. M^6^A reader proteins, such as YTH domain family 1/2/3 (YTHDF1/2/3), YTH domain-containing 1/2 (YTHDC1/2), heterogeneous nuclear ribonucleoprotein (HNRNP) family, and insulin-like growth factor 2 mRNA-binding protein (IGF2BP) family, recognize m^6^A-containing mRNA transcripts and perform diverse biological functions in the nucleus or cytoplasm.

Growing evidence shows that DNA methylation and histone modification, in part, controls AFB_1_-induced liver injury [7], whereas few investigations have uncovered the relationship between RNA methylation and AFB_1_-induced liver damage. N^6^-methyladenosine (M^6^A) RNA methylation is a conserved posttranslational modification that accounts for more than 60% of all RNA chemical modifications [8]. Dynamic and reversible m^6^A modification is coregulated by several proteins that can be classified into three groups: writers, erasers, and readers, which function as the addition, removal, and recognition of m^6^A modification sites (Figure 1). RNA methyltransferases, known as ‘writers’, are primarily composed of methyltransferase-like 3 (METTL3), methyltransferase-like 14 (METTL14), and Wilms’ tumor 1-associating protein (WTAP) [9]. The demethylases, can also be named ‘erasers’, consisting of fat mass and obesity-associated protein (FTO), and AlkB homolog 5 (ALKBH5) [10], which reverses m^6^A methylation. In addition, the biological function of m^6^A modification is modulated by m^6^A binding proteins, known as ‘readers’, to recognize the transcripts’ m^6^A sites. M^6^A reader proteins are predominantly in the YT521-B homology (YTH) protein families (YTH domain family 1/2/3 (YTHDF1/2/3) and YTH domain-containing 1/2 (YTHDC1/2)) [11]. A growing number of studies have identified that m^6^A modification plays an essential role in the regulation of biological processes, including mRNA splicing [12], nucleation [13], stability [14], and translation [15]. M^6^A modification is also closely associated with gene expression, stem cell differentiation, and cellular homeostasis balance [13,16]. The dysregulation of m^6^A patterns contributes to abnormal circadian rhythms, defective physiological functions, and aberrant nutritional metabolism, highlighting its role in obesity, hepatic diseases, and cancer [17,18,19]. Interestingly, emerging observations suggested that the m^6^A modification responds and adapts quickly to nutritional challenges, such as a high-fat diet or fasting, and supplementation of betaine, cycloleucine, and curcumin [20]. Thus, it is an interesting hotspot to explore the mechanism of how precisely m^6^A RNA methylation affects AFB_1_-induced liver damage.

Accumulating evidence indicates that some plant polyphenols could induce noteworthy antitoxic effect and may show resistance to AFB_1_-induced hepatotoxicity [21]. Resveratrol is a naturally-occurring polyphenolic compound found in many plants and food items, such as grapes, peanuts, and knotweed. Resveratrol has antioxidative, anti-inflammatory, antiapoptotic, and immunomodulatory effects [22], and the nature of resveratrol has been demonstrated to play a critical role in preventing liver damage, including acute alcoholic liver injury, hepatic ischemia-reperfusion injury, and hepatic fibrosis [23]. Furthermore, resveratrol regulates gene expression to exert its biological function, while the understanding of its regulatory mechanism is not sufficiently known. Importantly, resveratrol has been reported to be a potential epigenetic factor [24], and the current investigations indicated the regulatory role of resveratrol in DNA methylation [25] and histone modification [26], suggesting that resveratrol also probably exerts its hepatic protection function through regulating the modification of m^6^A.

The objective of this study was to investigate the effects of AFB_1_ and resveratrol on m^6^A RNA methylation, and their crosstalk in the regulation of hepatic function in mice, in order to provide a practical strategy for the treatment of liver disease and maintaining animal development.

## 2. Materials and Methods 

### 2.1. Animal Experiment Design

The experimental design and procedures in this study were conducted in conformity with the Institutional Animal Care and approved by the Committee of Nanjing Agricultural University (NJAU-CAST-2015-095) following the requirements of the Regulation for the Administration of Affairs Concerning Experimental Animals of China. Thirty-two C57BL/6J male mice (6 weeks of age) were purchased from the Yangzhou Institute of Experimental Animals. After two weeks of acclimation, the mice were randomly allocated to four groups of 8 mice (*n* = 8/group) as follows: the first group served as the control (CON) group, Groups 2, 3, 4 served as the resveratrol supplementation (RES) group, aflatoxin B_1_ supplementation (AFB_1_) group, and resveratrol supplementation in combination with aflatoxin B_1_ (ARE) group, respectively. The four groups were allowed a standard granulated diet (AIN-93 diet) [27]. During the entire 4-week experimental period, mice in the RES group were fed a standard diet supplemented with 500 mg/kg of resveratrol in pellet food according to Wang et al. [28] and Gordon et al. [29]. The AFB_1_ group was allowed a standard diet supplemented with 600 μg/kg of aflatoxin B_1_ [30], and the ARE group was treated with a standard diet supplemented with 500 mg/kg of resveratrol and 600 μg/kg of aflatoxin B_1_. All the diets were provided by Trophic Animal Feed High-Tech Co., Ltd. (Nantong, China). All the mice were housed at a temperature of 22 ± 1 °C, under a 12-h light cycle, with free access to water and food. In addition, mice body weights were measured weekly.

The resveratrol used in this experiment was purchased from Sigma–Aldrich (Merck Millipore, Darmstadt, Germany, CAS:501-36-0). The content of resveratrol was 99% as determined by HPLC analysis. The aflatoxin B_1_ standard (purity over 99%) used in this experiment was purchased from Beijing Solarbio Science&Technology Co., Ltd (Beijing, China)(CAS: SA8760).

### 2.2. Sample Collection

At 12 weeks of age, all mice were fasted overnight. Blood samples were collected by cardiac puncture technique following anesthesia with carbon dioxide. Blood samples were centrifuged at 4000 r/min for 10 min at 4 °C after being kept in room temperature for 30 min, and then serum obtained from the blood was stored at −80 °C for further determination. Liver tissues were immediately removed, thoroughly washed with phosphate-buffered saline (PBS), and then snap-frozen in liquid nitrogen and stored at −80 °C for further analysis. A portion of liver tissue was removed and fixed in formalin for histopathological examination.

### 2.3. Analysis of Serum Aminotransferase Activities

Activities of serum AST (CAS: C010-2-1) and ALT (CAS: C009-2-1) were measured using colorimetric assay kits (Nanjing Jiancheng Bioengineering Institute, Nanjing, China) by a microplate reader (Thermo Scientific, Wilmington, DE, USA) with a detection wavelength of 510 nm. All experimental procedures were performed according to the manufacturer’s protocol. 

### 2.4. Liver Histologic Evaluation

Liver tissues fixed in 10% neutral buffered formalin were dehydrated with a sequence of ethanol solutions and embedded in paraffin. 5-μm sections were cut, deparaffinized, rehydrated, and stained with hematoxylin-eosin (H&E). A light microscope was used (Nikon ECLIPSE 80i, Nikon Corporation, Tokyo, Japan) to evaluate and photograph the pathological changes. 

### 2.5. Detection of ROS

The levels of ROS were determined by dihydroethidium (DHE) staining in the liver. Briefly, cryosections from the snap-frozen liver (5 μm) were stained with ROS dye (Servicebio, Wuhan, China, CAS: GDP1018) and incubated at 37 °C in a light-proof incubator for 30 min. Subsequently, sections were incubated with DAPI in the dark for 10 min at room temperature, followed by washing with PBS three times. The sections were observed and photographed under a fluorescence microscope (LSM 700-Zeiss, Zeiss Corporation, Germany). An Image-Pro Plus 6.0 (Media Cybernetics, Rockville, MD, USA) software was used to quantify by measuring gray values. 

### 2.6. Analysis of Oxidative Stress Parameters

The liver sample (0.2 g) from −80 °C was suspended in ice-cold PBS (1.8 mL) and then homogenized using an Ultra-Turrax homogenizer (Tekmar, Ohio, USA) at 13,500× *g* for 1 min in ice-cold water. The homogenate was centrifuged at 3000× *g* for 15 min at 4 °C, and the supernatant was collected and analyzed quickly. All results were normalized to total protein concentrations in each sample for inter-sample comparisons. Malondialdehyde (MDA, Jiangsu, China, CAS: A003-1-1) and total antioxidant capacity (T-AOC, Jiangsu, China, CAS: A015-1-1) levels, catalase (CAT, Jiangsu, China, CAS: A007-1-1) activities, glutathione peroxidase activities (GSH-Px, Jiangsu, China, CAS: A005-1-1), and superoxide dismutase activities (SOD, Jiangsu, China, CAS: A001-2-2) were measured using commercial assay kits (Nanjing Jiancheng Bioengineering Institute) according to the manufacturer’s protocol. The levels of T-AOC, CAT, GSH-Px, and SOD were expressed as units (U) per milligram of protein. The level of MDA was expressed as nanomoles per milligram of protein.

### 2.7. Total RNA Extraction and Real-Time RT-PCR

Total RNA was isolated from snap-frozen liver tissues using TRIZol reagent (TaKaRa, Otsu, Shiga, Japan, CAS: 9108). The RNA concentration and absorbance at 260 and 280 nm, were quantified by Thermo NanoDrop 2000 Ultra Trace visible spectrophotometer (Thermo Fisher, Waltham, MA, USA). The RNA integrity was determined on 1% agarose gel with ethidium bromide staining. The mRNA was immediately reversed-transcribed into complementary DNA (cDNA) using the PrimerScript RT reagent kit (TaKaRa, Otsu, Shiga, Japan, CAS: RR036A) according to the manufacturer’s protocol. Real-time PCR was conducted in the ABI StepOnePlus^TM^ PCR system. The primer sequences are listed in Table 1 and synthesized by Sangon Biotech Co. Ltd. (Shanghai, China). PCR reaction mixture of 20 μL was prepared using 0.4 μL each of forward and reverse primers, 0.4 μL of 50× ROX Reference Dye 2, 10 μL of 2× ChamQ SYBR qPCR Master Mix (Vazyme Biotechnology, Nanjing, China, CAS: Q311-02), 6.8 μL of double-distilled H_2_O and 2 μL cDNA. The following thermal condition was used for qRT-PCR: 3 min at 95 °C, 40 cycles of 10 sec at 95 °C, and 30 sec at 60 °C. The relative mRNA expression was calculated by the 2^−ΔΔCt^ method after normalization with housekeeping genes GAPDH. Samples in the CON group were used as calibrators. The sequences of primers used in this experiment are shown in Table 1.

### 2.8. Measurement of Total m^6^A

Total m^6^A levels in mRNA were determined in 20 ng aliquots of mRNA extracted from liver tissues using an EpiQuik^TM^ m^6^A RNA methylation quantification kit (Epigentek; Wuhan, China, CAT. No. p-9005). Total RNA was bound to strip wells using RNA high binding solution. M^6^A was conducted using capture and detection antibodies. The detected signal was enhanced and then quantified colorimetrically via reading the absorbance in a microplate spectrophotometer (Thermo Fisher, Waltham, MA, USA). The m^6^A level was calculated by OD intensity. 

### 2.9. Western Blotting

The liver sample (20 mg) was suspended in RIPA buffer (200 μL) (Beyotime Biotechnology, P0013B) with protease and phosphatase inhibitor cocktail (Beyotime Biotechnology, P1045), and then homogenized using the glass homogenizer on ice. The homogenate was centrifuged at 12,000× *g* for 5 min at 4 °C, and the supernatant was collected. The protein concentration in the supernatant was determined using a bicinchoninic acid (BCA) kit (Beyotime Biotechnology, CAS: P0012). Samples (30 μg of protein) were mixed with 5× sample buffer and boiled at 100 °C for 10 min. The protein samples were separated on 12% SDS-PAGE gels and electrotransferred onto an immobile membrane (PVDF membrane, Merck Millipore, Darmstadt, Germany, CAS: IPVH00010) with transfer buffer. The membranes were blocked at room temperature with 5% non-fat dry milk in TBST (0.05% Tween-20, 100mmol/L Tris- HCL, and 150 mmol/L NaCl, pH 8.0) for 2 h. After blocking, the membranes were incubated overnight with primary antibodies at 4 °C. After washing three times with TBST, the blots were incubated with a 1:7500-dilution of goat anti-mouse or anti-rabbit HRP-conjugated secondary antibodies (Abcam, ab205718 or ab205719) for 90 min at room temperature. The blots were visualized using the enhanced chemiluminescence kit (Merck Millipore, Darmstadt, Germany, CAS: WBKLS0500), followed by autoradiography. Images were recorded by a luminescence image analyzer LAS-4000 system (Fujifilm Co. Ltd., Tokyo, Japan) and were quantified by Image-Pro Plus 6.0 (Media Cybernetics, Rockville, MD, USA). ACTB antibody was used as the internal standard to normalize the signals. Primary antibodies used in the experiment are listed in Table 2. 

### 2.10. Statistical Analysis

Data were expressed as means with SEM (standard error of the mean) and analyzed by the two-way ANOVA. The classification variables were dietary resveratrol supplementation (CON + AFB_1_ × RES + ARE), dietary aflatoxin B_1_ supplementation (CON + RES × AFB_1_ + ARE), and their interaction (CON × RES × AFB_1_ × ARE). Duncan’s multiple range test was used to determine the differences between the four groups when a statistically significant resveratrol × aflatoxin B_1_ interaction was observed. The SPSS 25.0 (SPSS Inc, Chicago, IL, USA) was used to analyze these results. *p* < 0.05 was considered statistically significant, and *p* < 0.01 was considered very significant.

## 3. Results

### 3.1. Growth Analysis

**Figure 2:**
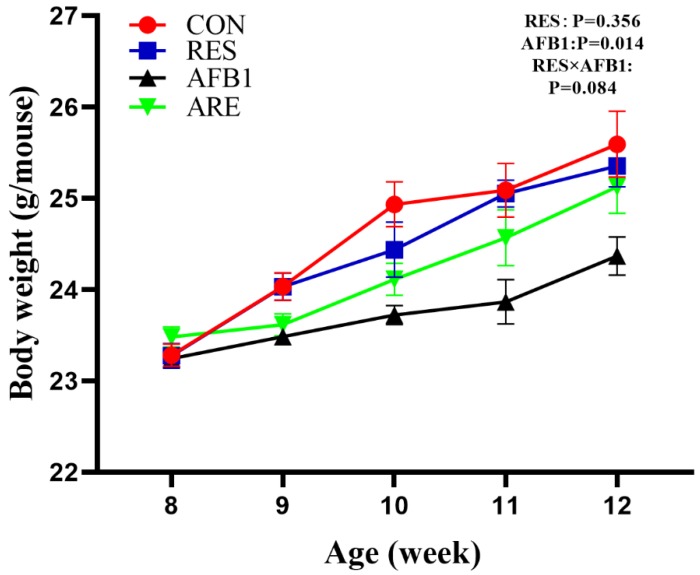
The effect of resveratrol on AFB_1_-induced body weight gain in mice. The body weights were recorded every week. CON, basal diet; RES, basal diet + 500 mg/kg resveratrol. AFB_1_, basal diet + 600 μg/kg aflatoxin B_1_; ARE, basal diet + resveratrol (500 mg/kg) and aflatoxin B1 (600 μg/kg). All data were analyzed using two-way ANOVA. Data are represented as mean ± SEM, *n* = 8.

During the entire 4 weeks period, the body weight of mice in the AFB_1_ group was consistently lower than the other three groups. Dietary resveratrol supplementation (the ARE group) increased body weight and improved growth performance compared to the AFB_1_ group at 12 weeks of age (*p* < 0.05; Figure 2). There were no differences in body weight between control and RES groups.

### 3.2. Activities of Serum Aspartate Aminotransferase and Alanine Aminotransferase

We next determined the activities of serum ALT and AST (Table 3). Compared with the CON group, the activities of serum ALT and AST were significantly increased (ALT, *p* < 0.05; AST, *p* < 0.01) in the AFB_1_ group. We also noted that the activities of serum ALT and AST in the ARE group were markedly lower than the AFB_1_ group (ALT, *p* < 0.05; AST, *p* < 0.01). In addition, no changes were observed between the CON group and the RES group.

### 3.3. Liver Histological Changes

**Figure 3:**
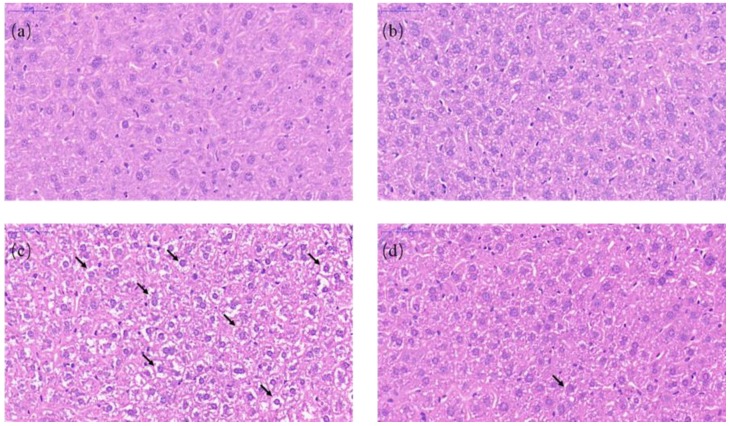
Light microscopy of liver tissues in different groups (40× magnification). (**a**) CON, (**b**) RESl., (**c**) AFB_1_, and (**d)** ARE. Hematoxylin–eosin staining of liver section. Scale bars, 50 μm.

We next performed the staining of hematoxylin–eosin to observe the histopathological changes in the liver. Normal histological structures were discovered in the liver of the CON group and the RES group (Figure 3a,b). In the liver sections of the AFB_1_ group, we observed that vacuolization and cell edema were extremely prevalent in the hepatocytes (Figure 3c). Compared with the AFB_1_ group, vacuolization and cell edema were significantly decreased in the ARE group (Figure 3d). The arrows showed vacuolization and cell edema.

### 3.4. ROS Content

**Figure 4:**
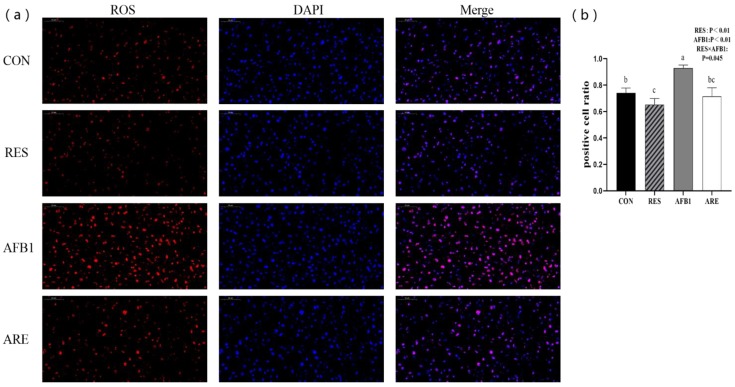
Analysis of ROS content in different groups (40× magnification). (**a**) Representative pictures of ROS detection using dihydroethidium (DHE)-stained liver cryosections in mice. Scale bars, 50 μm. (**b**) Quantification of ROS content was performed by measuring gray values using Image-Pro Plus 6.0 software. (*n* = 3 per genotype). All data were analyzed by using two-way ANOVA and Duncan’s post hoc testing, where appropriate. Data are represented as mean ± SEM, *n* = 3. *^a^*^–^*^c^* Mean values within a line with different superscript letters were significantly different (*p* < 0.05).

In the present study, we found that ROS content in the AFB_1_ group was significantly higher than the CON group (*p* < 0.05). The ARE group showed lower content of ROS compared with the AFB_1_ group (*p* < 0.05). Interestingly, we also noted that dietary resveratrol supplementation could notably scavenge ROS in the RES group relative to the CON group (*p* < 0.05) (Figure 4a). Quantification of ROS content in different groups is shown in Figure 4b. 

### 3.5. Hepatic Redox Status

We next determined the activities of antioxidant enzymes (GSH-PX, CAT, and SOD) and the levels of lipid peroxidation (MDA) and antioxidant capacity (T-AOC) in the liver. The data are shown in Table 4. Compared to the CON group, the AFB_1_ group showed up-regulated concentration of MDA (*p* < 0.05), decreased activity of CAT (*p* < 0.05), and lower level of T-AOC (*p* < 0.05). Moreover, the ARE significantly reduced the content of MDA (*p* < 0.05), markedly increased the activity of CAT (*p* < 0.05), and the level of T-AOC (*p* < 0.05) relative to the AFB_1_. We also noted that mice given aflatoxin B_1_ (the AFB_1_ group and the ARE group) showed higher content of MDA (*p* < 0.01), lower activities of CAT and SOD (*p* < 0.01), and less level of T-AOC (*p* < 0.01) compared to mice fed basal diet without aflatoxin B_1_. No changes were observed in the content of MDA, the level of T-AOC, and the activities of CAT, GSH-PX, and SOD between the CON group and the RES group (*p* > 0.05). In addition, there were no changes in the activity of GSH-PX in different groups (*p* > 0.05).

### 3.6. Hepatic Antioxidant Gene Expression

**Figure 5:**
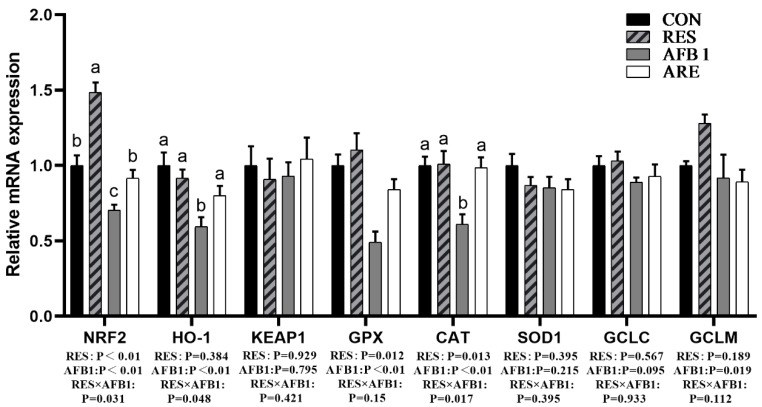
The effect of resveratrol on the hepatic antioxidant parameters in AFB_1_-challenged mice. qPCR analysis of hepatic antioxidant mRNA expression in different groups (*n* = 6/group). All data were analyzed using two-way ANOVA and Duncan’s post hoc testing, where appropriate. Data are represented as mean ± SEM. ^a^^–^^c^ Mean values within a line with different superscript letters were significantly different (*p* < 0.05).

We next determined the mRNA expression of antioxidant genes in the liver. The data of mRNA expression are shown in Figure 5 The mRNA expression of genes involved in oxidative stress, including Nrf2, HO-1, GPX, and CAT, were dramatically declined in the liver of the AFB_1_ group as compared with the CON group (*p* < 0.05). The expression of Nrf2, HO-1, GPX, and CAT mRNA in the liver of the ARE group were significantly elevated relative to the AFB_1_ group (*p* < 0.05). No changes were observed in Keap1, SOD1, and GCLC mRNA among the four groups (*p* > 0.05).

### 3.7. Hepatic Apoptosis Gene Expression

**Figure 6:**
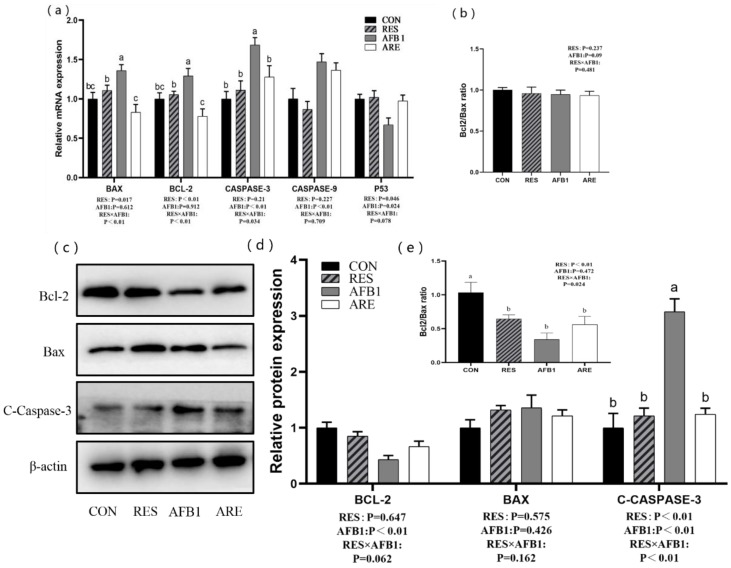
The effect of resveratrol on the hepatic apoptosis in AFB_1_-challenged mice. (**a**) qPCR analysis of hepatic apoptosis mRNA expression in different groups (*n* = 6/group). (**b**) Analysis of *bcl-2/bax* mRNA expression ratio in different groups (*n* = 6/group). (**c**) Immunoblot analysis of hepatic apoptosis protein expression in different groups (*n* = 3/group). (**d**) Quantification of apoptosis protein expression was performed by measuring gray values using Image-Pro Plus 6.0 software (*n* = 3/group). (**e**) Analysis of bcl-2/bax protein expression ratio in different groups (*n* = 3/group). All data were analyzed by using two-way ANOVA and Duncan’s post hoc testing, where appropriate. Data are represented as mean ± SEM. ^a^^–^^c^ Mean values within a line with different superscript letters were significantly different (*p* < 0.05). (Bcl-2, 26 kDa; Bax, 21 kDa; cleaved-Caspase-3, 17 kDa; β-actin, 42 kDa).

We determined the mRNA and protein levels of apoptosis genes in the liver. qRT-PCR results are shown in Figure 5. Compared to the CON group, the expression of Bax, Bcl-2, and caspase-3 mRNA were significantly increased (*p* < 0.05) in the AFB_1_ group. There was a significant reduction (*p* < 0.05) in the mRNA expression of Bax, Bcl-2, and caspase-3 in the liver of the ARE group compared to the AFB_1_ group. However, the differences in the ratio of bcl-2/bax mRNA expression were not observed between the four groups (Figure 6b). In addition, the expression of caspase-9 mRNA in the liver of mice given aflatoxin B_1_ (the AFB_1_ group and the ARE group) was improved compared with that in the both CON and RES group (*p* < 0.01) (Figure 6a). Western blot analysis revealed that AFB_1_ markedly up-regulated the protein expression of caspase-3 compared with the CON (*p* < 0.05), and the ARE could reverse this elevation (*p* < 0.05). The mice from the AFB_1_ and ARE group exhibited lower protein expression of Bcl-2 than the mice from the CON and RES group (*p* < 0.01), while no changes were observed in Bax expression between the four groups (*p* > 0.05) (Figure 6c,d). Noticeably, the ratio of bcl-2/bax protein expression showed significantly decrease (*p* < 0.05) in the AFB_1_ group compared with the CON group, whereas no difference was found among the RES, AFB_1_, and ARE group (Figure 6e).

### 3.8. Levels of m^6^A RNA Methylation

**Figure 7:**
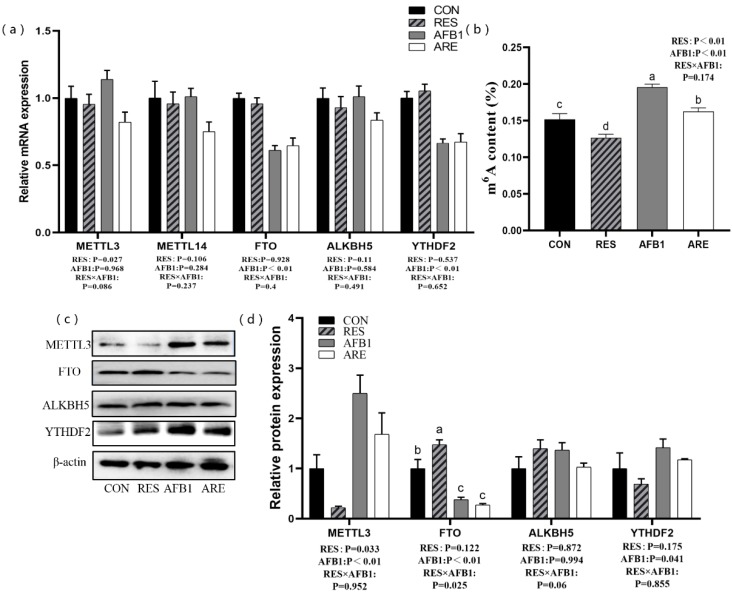
The effect of resveratrol on m^6^A RNA methylation in AFB_1_-challenged mice. (**a**) qPCR analysis of m^6^A-related mRNA expression in different groups (*n* = 6/group). (**b**) Measurement of m^6^A content in different groups (*n* = 4/group). (**c**) Immunoblot analysis of m^6^A-related protein expression in different groups (*n* = 3/group). (**d**) Quantification of m^6^A-related protein expression was performed by measuring gray values using Image-Pro Plus 6.0 software (*n* = 3/group). CON, basal diet; RES, basal diet + 500 mg/kg resveratrol. AFB_1_, basal diet + 600 μg/kg aflatoxin B_1_; ARE, basal diet + resveratrol (500 mg/kg) and aflatoxin B_1_ (600 μg/kg). All data were analyzed using two-way ANOVA and Duncan’s post hoc testing, where appropriate. Data are represented as mean ± SEM. ^a^^–^^c^ Mean values within a line with different superscript letters were significantly different (*p* < 0.05). (METTL3, 65–70 kDa; FTO, 58 kDa; ALKBH5, 40–50 kDa; YTHDF2, 62 kDa; β-actin, 42 kDa).

We next determined the level of m^6^A modification and the expression of m^6^A-related genes and proteins in the liver. The mice from the AFB_1_ and the ARE group exhibited lower mRNA expression of FTO and YTHDF2 than the mice from the CON and the RES group (*p* < 0.01) (Figure 7a). Compared with the CON group, the expression of FTO protein was remarkably decreased in the AFB_1_ group (*p* < 0.05). Mice given aflatoxin B_1_ (the AFB_1_ group, the ARE group) showed a high level of METTL3 than the other mice (the CON group, the RES group) (*p* < 0.01) (Figure 7c,d). Interestingly, we also noted that the RES significantly down-regulated the expression of METTL3 protein, while dramatically increased the expression of FTO protein (*p* < 0.05) compared with the CON (Figure 7d). In addition, higher content of m^6^A was observed in the AFB_1_ group than the CON group (*p* < 0.05). On the contrary, the RES group exhibited the lower level of m^6^A than the CON group (*p* < 0.05), and the ARE could attenuate the ascending level of m^6^A relative to the AFB_1_ (*p* < 0.05) (Figure 7b).

## 4. Discussion

AFB_1_ inducing the dynamic changes of hepatic gene expression at the post-transcriptional level remain largely unknown. Furthermore, resveratrol exerts an antitoxic role in the liver, however, its precise mechanism is still not sufficiently known. The current study provided evidence of the protective potential of resveratrol against the AFB_1_-induced liver damage in mice. Dietary resveratrol supplementation exerted several powerful effects, including a decrease of ROS concentration, alleviation of oxidative stress, inhibition of apoptosis, and the down-regulation of m^6^A level. Besides, the hepatic function damage by AFB_1_ might be due to the increase of m^6^A. Thus, we suggested that m^6^A RNA methylation may involve in AFB_1_-induced hepatotoxicity, and dietary resveratrol supplementation can reverse m^6^A level in the liver, then regulate the expression of hepatic antioxidant and apoptosis genes, and eventually repair hepatic function.

It is worth noting that the network of DNA methylation and histone modification, in part, regulates AFB_1_-induced liver injury [7], whereas few investigations have uncovered the crosstalk between RNA methylation and AFB_1_-induced liver damage. M^6^A is the most common prevalent internal RNA methylation modification that exerts its biological functions, including the regulation of mRNA splicing, export, localization, stability, and translation [20,31], and regulates gene expression. Emerging evidence indicates that the dynamic and reversible nature of m^6^A modification plays a critical role in nutritional physiology and metabolism [20]. In this study, we found that AFB_1_ significantly increased the protein expression of METTL3, whereas it markedly reduced the expression of FTO in the liver, and increased the level of m^6^A. Notably, the cell apoptosis was significantly increased in AFB_1_-treated mice with an elevation of *bax* mRNA, a decrease of bcl-2 protein, and the declining tendency of bcl-2/bax protein expression ratio. Bcl-2 and bax play an antagonistic role in maintaining the apoptosis process. Bcl-2 is the core element that performs the role of resistance in apoptosis, whereas bax functions as a promoter of apoptosis [32,33], and cellular homeostasis depends on the balance of the bcl-2/bax ratio. A recent study demonstrated that m^6^A modification promotes the translation of *bcl-2* mRNA in the human acute myeloid leukemia MOLM-13 cell line [34]. This observation implicated the potential regulatory role of m^6^A modification in the cell apoptosis to be associated with the translation of bcl-2 mRNA. In contrast, here, we found that an elevated mRNA expression, but a decreased protein expression of Bcl-2 in the AFB_1_ group. We suspect that this result may be due to enhanced transcriptional level while translation efficiency decreased, and the exact mechanism needs further investigation. Furthermore, previous investigations reported that cells undergo the apoptosis process via a caspase-independent or caspase-dependent pathway [35]. Supportively, an increase of *caspase-3* mRNA and cleaved-caspase-3 protein were observed in AFB_1_ treatment. Caspase-3 is one of the cysteine proteases which plays a critical role in the execution of apoptosis. Apoptosis signal could lead to the activation of caspase-3 and formulate cleaved-caspase-3. The level of cleaved-caspase-3 directly reflects the degree of apoptosis [36]. Interestingly, a novel study supported that silencing METTL3 could inhibit apoptosis in hypoxia/reoxygenation-treated cardiomyocytes [37]. Overexpression of METTL3 or knockdown of FTO enhanced m^6^A levels and activated apoptosis in cisplatin-treated human kidney proximal tubular cells [38]. Conversely, METTL3 knockdown could active caspase-3 in gastric cancer cells [39]. This evidence suggests that m^6^A modification participates in the regulation of the apoptotic pathway. Taken together, both our findings and above investigations hint that the relationships between AFB_1_-induced hepatic apoptosis and m^6^A RNA methylation is robust, and m^6^A modification may participate in the apoptotic process through the regulation of the caspase-3-dependent pathway.

Growing observations have supported that resveratrol exerts a strong antitoxic effect [40,41,42]. Our data revealed that dietary resveratrol supplementation repaired defective hepatic structure and reversed liver damage caused by oxidative stress. The antioxidant property of resveratrol has been considered to be principally associated with its capacity in scavenging free radicals [22]. Interestingly, growing observations showed that the antiapoptotic effect of resveratrol is involved in Fas signaling-dependent apoptosis signal, which directly mediate the cleavage of downstream effector such as caspase-3 [43]. However, the inhibition of the antiapoptotic protein of the bcl-2 family, and activation of the pro-apoptotic protein of bax by resveratrol have also been reported to have the regulatory role in caspase-dependent signaling [44]. In this study, dietary resveratrol supplementation suppressed cell apoptosis via decreasing protein expression of cleaved-caspase-3 in the AFB_1_-damaged mice, which is consistent with the previous studies [45], whereas no changes were observed in bcl-2/bax protein expression ratio between the AFB_1_ group and the ARE group. Thus, we speculated that the underlying network of the protective role of resveratrol in AFB_1_-induced hepatotoxicity is associated with Fas-mediated apoptosis signal instead of changing the proteins of the bcl-2 family, and this speculation still needs further confirmation. These results highlight the effective protection of resveratrol in AFB_1_-induced liver injury. Fascinatingly, emerging observations indicated that nutritional challenges, such as a high-fat diet, a dietary fasting state, and dietary supplement with betaine, cycloleucine, and curcumin [20,46] regulate the gene expression by m^6^A RNA methylation. Here, we also found that dietary resveratrol supplementation in AFB_1_-treated mice significantly reduced the level of m^6^A compared with the AFB_1_ group. In addition, mice in the RES group exhibited a significant reduction of METTL3 protein expression and a prominent increase of FTO protein expression. Consistent with our previous study, resveratrol was able to reduce the abundance of m^6^A modification in piglets [47]. Therefore, these data suggest that the protective function of resveratrol against AFB_1_-induced liver damage is related to the reduction of m^6^A modification.

However, the precise mechanisms of AFB_1_ and the regulatory role of resveratrol on m^6^A RNA methylation need to be further explored. Our previous study demonstrated that disruption of circadian rhythms results in high levels of ROS in the liver and increased METTL3, followed by the up-regulation of m^6^A modification [17]. H_2_O_2_ treatment in HepG2 cells and acetaminophen (APAP) injection in WT mice verified that ROS enormously increased the abundance of m^6^A [17]. These findings confirmed that ROS significantly impacts m^6^A RNA methylation. In the present study, we also found that AFB_1_-treated mice significantly prompted ROS accumulation and increased the level of m^6^A modification. Thus, it is possible that the increase of m^6^A induced by AFB_1_ is related to the accumulation of ROS in the liver and eventually causes liver injury. Furthermore, we also found that dietary resveratrol supplementation in AFB_1_-treated mice significantly reduced the ROS concentration and decreased the abundance of m^6^A modification compared with the AFB_1_ group. It is well known that the protective role of resveratrol is associated with its ability to remove ROS in the liver [48]. Therefore, we considered that resveratrol scavenges the ROS and decreases the hepatic m^6^A level in AFB_1_-treated mice, eventually improving liver function. 

## 5. Conclusions

We found the role of m^6^A modification on the potential mechanism of AFB_1_-induced hepatotoxicity. Mechanistically, AFB_1_-induced ROS accumulation changed m^6^A modification. We also discovered the protective role of resveratrol in alleviating hepatic disorder induced by AFB_1_ may be due to the removal of ROS, followed by the decreased abundance of m^6^A modification. Together, this work provides key insights into the potential avenues for the prevention and treatment of the adverse effects of ROS accumulation related to chronic liver diseases and even cancer.

## Figures and Tables

**Table 1:** Primer sequences used for quantitative real-time PCR in this study.

Primers ^1^	Accession No.	Sequences (F/R, 5′-3′)
*NRF2*	NM_010902	GGTTGCCCACATTCCCAAAC
		AGTGACTGACTGATGGCAGC
*HO-1*	NM_010442	GTCAGGTGTCCAGAGAAGGC
		CATCACCTGCAGCTCCTCAA
*KEAP1*	NM_001110307	AAGTGTGAGATCCTGCAGGC
		CGACTAGATGCCACTCGTCC
*GPX1*	NM_001329528	TGAACGATCTGCAGAAGCGT
		TAGGAGTTGCCAGACTGCTG
*CAT*	NM_009804	TTCGTCCCGAGTCTCTCCAT
		GAGTGTCCGGGTAGGCAAAA
*GCLC*	NM_010295	TACCGAGGCTACGTGTCAGA
		TCTCGTCAACCTTGGACAGC
*GCLM*	NM_008129	GAATGCACCATGTCCCATGC
		CGATGACCGAGTACCTCAGC
*SOD1*	NM_011434	GGAACCATCCACTTCGAGCA
		CCAATCACTCCACAGGCCAA
*BAX*	NM_007527	GGTGGCAGCTGACATGTTTG
		TTAGTGCACAGGGCCTTGAG
*BCL-2*	NM_009741	CTTCTCTCGTCGCTACCGTC
		CAATCCTCCCCCAGTTCACC
*CASP-3*	NM_001284409	ACATGGGAGCAAGTCAGTGG
		CCGTACCAGAGCGAGATGAC
*CASP-9*	AB019600	GTCACAGACCTTGAGACCCG
		GGCAGTCAGGTCGTTCTTCA
*P53*	AB020317	TGCATGGACGATCTGTTGCT
		GTGGTATACTCAGAGCCGGC
*METTL3*	NM_019721	ACCACAACAGCCAAGGAACA
		CCAATTCCATGGCCCTTCCT
*METTL14*	NM_201638	TATGCTTGCGAAAGTGGGGT
		CCACCTCTCTCTCCTCGGAA
*FTO*	NM_011936	GATGACCTCAATGCCACCCA
		ACTAAACCGAGGCTGTGAGC
*ALKBH5*	NM_172943	GTCCCGGGACAACTACAAGG
		TATTTCCGCTTGGTGGTCCC
*YTHDF2*	NM_145393	CAGCTCTCAGTCCAGCAACA
		AGTAGATCCAGAACCCGCCT
*GAPDH*	NM_008084	TTCACCACCATGGAGAAGGC
		TGAAGTCGCAGGAGACAACC

^1^ NRF2, nuclear factor, erythroid 2 like 2; HO-1, heme oxygenase 1; KEAP1, kelch like ECH associated protein 1; GPX1, glutathione peroxidase 1; CAT, catalase; GCLC, glutamate-cysteine ligase catalytic subunit; GCLM, glutamate-cysteine ligase modifier subunit; SOD1, superoxide dismutase 1; BAX, BCL2 associated X; BCL-2, B cell lymphoma 2; CASP-3, caspase 3; CASP-9, caspase 9; METTL3, methyltransferase-like 3; METTL14, methyltransferase-like 14; FTO, fat mass and obesity-associated protein; ALKBH5, AlkB homolog 5; YTHDF2, YTH domain family 2; GAPDH, glyceraldehyde-3-phosphate dehydrogenase.

**Table 2:** The information of antibodies used in this study.

Antibodies	Identifier	Source	Host
BCL-2	12789-1-AP	Proteintech	Rabbit
BAX	50599-2-Ig	Proteintech	Rabbit
CASPASE-3	19677-1-AP	Proteintech	Rabbit
METTL3	ab240595	Abcam	Rabbit
FTO	27226-1-AP	Proteintech	Rabbit
ALKBH5	16837-1-AP	Proteintech	Rabbit
YTHDF2	24744-1-AP	Proteintech	Rabbit
ACTB	60008-1-Ig	Proteintech	Mouse

**Table 3:** The effect of dietary resveratrol supplementation on the activities of ALT and AST in the liver of AFB_1_-challenged mice.

Items	Experiment Groups				SEM	*p*		
	CON	RES	AFB_1_	ARE		A	R	A × R
ALT (U/L)	9.01 ^bc^	8.24 ^c^	12.43 ^a^	10.01 ^b^	1.89	<0.01	<0.01	0.034
AST (U/L)	9.23 ^c^	11.70 ^c^	21.21 ^a^	14.83 ^b^	5.22	<0.01	0.048	<0.01

Data are expressed as mean ± SEM, *n* = 8. ALT, alanine aminotransferase; AST, aspartate aminotransferase. CON, basal diet; RES, basal diet with dietary resveratrol (500 mg/kg) supplementation. AFB_1_, basal diet with dietary aflatoxin B_1_ (600 μg/kg) supplementation; ARE, basal diet with dietary resveratrol (500 mg/kg) and aflatoxin B_1_ (600 μg/kg) supplementation. A, dietary aflatoxin B_1_ supplementation; R, dietary resveratrol supplementation; A×R, interaction between the corresponding parameters. Data were analyzed using two-way ANOVA and Duncan’s post hoc testing, where appropriate. ^a^^–^^c^ Mean values within a line with different superscript letters were significantly different (*p* < 0.05).

**Table 4:** The effect of dietary resveratrol supplementation on the hepatic redox status in AFB_1_-challenged mice.

Items	Experiment Groups				SEM	*p*		
	CON	RES	AFB_1_	ARE		A	R	A × R
MDA (nmol/mgprot)	3.99 ^bc^	3.74 ^c^	5.04 ^a^	4.22 ^b^	0.61	<0.01	<0.01	0.036
CAT (U/mgprot)	79.54 ^a^	76.70 ^ab^	67.12 ^c^	72.97 ^b^	7.175	<0.01	0.46	0.04
GSH-PX (U/mgprot)	939.02	936.73	875.81	921.17	60.584	0.064	0.3	0.252
SOD (U/mgprot)	779.20	755.83	653.81	694.52	22.420	<0.01	0.589	0.053
T-AOC (U/mgprot)	0.67 ^a^	0.70 ^a^	0.39 ^c^	0.56 ^b^	0.086	<0.01	<0.01	0.025

Data are expressed as mean ± SEM, *n* = 8. MDA, malondialdehyde; CAT, catalase; GSH-PX, glutathione peroxidase; SOD, superoxide dismutase; T-AOC, total antioxidant capacity. A, dietary aflatoxin B1 supplementation; R, dietary resveratrol supplementation; A × R, interaction between the corresponding parameters.Data were analyzed using two-way ANOVA and Duncan’s post hoc testing, where appropriate. ^a^^–^^c^ Mean values within a line with different superscript letters were significantly different (*p* < 0.05).
